# Local Radiation Therapy for Palliation in Patients With Multiple Myeloma of the Spine

**DOI:** 10.3389/fonc.2019.00601

**Published:** 2019-07-03

**Authors:** Daniel Mark, Philip Gilbo, Raymond Meshrekey, Maged Ghaly

**Affiliations:** ^1^Department of Radiation Medicine, Northwell Health, Lake Success, NY, United States; ^2^New York Institute of Technology College of Osteopathic Medicine, Glen Head, NY, United States

**Keywords:** multiple myeloma, radiotherapy, spine, palliation, pain

## Abstract

**Purpose:** The objective of this study was to assess a contemporary cohort of patients with multiple myeloma referred for palliative radiation to the mobile spine for clinical and radiological responses.

**Materials/Methods:** The records of patients treated between 2009 and 2016 with radiotherapy for multiple myeloma of the spine were retrospectively reviewed. Demographics, systemic therapy, radiation dose, number of fractions, radiographic response based upon adapted RECIST criteria, and symptomatic response were recorded.

**Results:** Eighty eight patients and 98 treatment courses were analyzed. All courses were analyzed for symptomatic response and 61 of the treatment courses were available for radiologic follow-up. The median follow-up was 9.7 months with a median radiation dose of 25 Gy (12.5–50 Gy) delivered in a median of 10 fractions (5–25 fractions). Fifty-four percent of patients had a high-risk lesion. Symptomatic response as measured by a decrease of ≤5 points on the pain related scale was 83% and 34% of patients had a decrease of >5 points. Of 35% of patients that had neurologic impairments prior to treatment, improvement was identified 83% of the time. Radiographic response was noted as 13% complete response, 16% partial response, 57% stable disease, and 13% disease progression. Specifically, high-risk lesions treated with radiation alone demonstrated no regression with only 10% demonstrating partial response.

**Conclusion:** This retrospective series of patients treated with palliative intent for multiple myeloma using various dose and fractionation schemes showed favorable symptomatic relief in most patients. Radiographic response did not correlate with clinical response with fewer patients having radiologic disease regression. Longer follow-up is necessary to determine if the lack of radiologic response is associated with clinically relevant recurrent pain.

## Introduction

Multiple myeloma is a relatively rare malignancy, with ~30,770 cases diagnosed per year and 12,770 deaths per year in the United States as of 2018 (1). It is a neoplasm arising from plasma cells, post-germinal B cells, which progresses to end-organ damage, usually involving the bone marrow and involving the bone in 80% of cases (2, 3). Given its systemic nature, chemotherapy is the main treatment modality. However, as the disease involves the bone marrow/bone, lesions may progress and cause bone pain, fractures, spinal cord/nerve compression, and may be associated with soft-tissue masses posing a higher risk to patients. These local lesions require more local therapy such as surgical excision and/or radiotherapy for symptomatic relief and/or to prevent further local progression (4). Lesions in the mobile spine are of particular concern given the proximity to the spinal cord/cauda equina. The mobile spine is fundamental to mobility and progression of lesion(s) can lead to a significant deterioration of quality of life (5). Previous retrospective studies have noted that local pain control was positively related to applied radiation dose (6) and others have analyzed surgery on spinal multiple myelomatous lesions noting that patients undergoing surgery experienced pain relief as well (7, 8).

In this study we sought to analyze our institutional experience with treatment of multiple myeloma of the mobile spine with radiotherapy to assess both radiological and symptomatic outcomes.

## Materials and Methods

### Patient Inclusion

We retrospectively reviewed the medical records of patients who were treated for pathologically confirmed multiple myeloma of the spine treated with palliative intent conventional radiotherapy between 2009 and 2016 within our multi-center institution. The study was approved by our institutional review board.

### Data Recording

We recorded demographic data including age and sex, information regarding the patients' spinal myelomatous lesion(s), neurological symptomatology before and after treatment, systemic therapy use, details regarding radiation including dose, fractionation, duration and modality, including potential incidences of re-irradiation and recorded radiographic response based on available imaging and clinical response based on patient reported pain scores. High-risk lesions were defined as those with compression fracture(s), cord compression, or having a paraspinal soft tissue mass component.

We excluded patients who received a single fraction of radiotherapy and stereotactic body radiation therapy. We separately assessed patients' radiological response assessment from that cohort with documented radiographic imaging available.

### Data Interpretation

We utilized the most detailed form of imaging and the latest/most recent imaging to assess one of the primary endpoints of radiographic response—MRI if available, followed by CT or PET and X-ray or bone scan if other studies were unavailable given that the latter studies may show less response due to delayed time in bone healing compared to PET/CT or MRI. We determined radiographic response based on adapted RECIST criteria. Possible response included complete response (no lesion visible), partial response (improvement of diffuse infiltration or reduction in size of the focal lesion), stable disease, or progression (aggravation of the diffuse bone marrow infiltration or increase in size of the focal lesion) (9).

In terms of symptomatic response, we utilized a 0–10 numeric pain rating scale (NPRS) and defined some pain relief as less than or equal to a decrease of 5 and significant pain relief as greater than a decrease of 5 on the NPRS. This score was recorded from the last known follow-up for the patient.

Treatment dose and fractionation was noted and we compared doses for radiographic response categories utilizing a one-way ANOVA test and symptomatic responses utilizing a two-sample, two-tailed *t*-test.

## Results

### Patient Characteristics

Patient and treatment characteristics are summarized in Table 1.

**Table 1:** Patient characteristics.

**Patient Characteristics**	***N* (%) or [range]**
Number of patients	88
Male Female	46 (52%) 42 (48%)
Median age	65 [43–98]
Number of vertebral segments treated	98
Known systemic therapy administration	83 (94%)
Number of sites with radiographic follow-up	61 (62%)
Median number of radiotherapy fractions	10 [5–25]
Median radiologic follow-up	9.7 months [0.1–107.5 months]
High-risk features	33 of 61 (54%)
Cord compression Compression fractures Paraspinal soft tissue component	8 19 6

A total of 88 patients and 98 vertebral segments treated with palliative radiotherapy were retrospectively reviewed. Of the 98 treatment courses, 61 (62%) had appropriate follow-up imaging to assess radiological response. Median follow-up for these patients, defined as time from date of last fraction of radiotherapy to time of latest radiological exam was 9.7 months (0.1–107.5 months). Thirty three of the 61 treatment-courses were for high-risk lesions including 8 treatments for cord compression, 19 treatments for compression fractures, and 6 treatments for lesions with a paraspinal soft tissue mass component.

The median number of fractions administered was 10 (mean 9.8, range 5–25), and median radiation dose administered was 25 (mean 27.1 Gy, range 12.5–50 Gy). The three most common fractionation schemes were 30 Gy in 10 fractions (*n* = 16), 20 Gy in 5 fractions (*n* = 13), and 24 Gy in 8 fractions (*n* = 10).

Systemic therapy was documented in 83 of the 88 patients (94%) and included chemotherapy, steroids, and/or stem cell transplant within several weeks their radiotherapy course. For 5 patients, chemotherapy was either refused or no records were available.

### Radiological Response

Table 2 summarizes radiological treatment responses in all patients and also stratifies those with and without high-risk features.

**Table 2:** Radiological response.

**Response characteristics**	***N* (%)**
Adapted RECIST criteria radiological response (*n* = 61)	
Complete response	8 (13%)
Partial response	10 (16%)
Stable disease	35 (57%)
Progression	8 (13%)
Patients with high-risk features (*n* = 33)	
Radiotherapy alone	
Complete response	0 (0%)
Partial response	3 (9%)
Stable disease	25 (76%)
Progression	2 (6%)
Surgery+RT^*^ with complete/partial response	3 (9%)
Patients without high-risk features (*n* = 28)Radiotherapy alone	
Complete response Partial response Stable disease Progression Surgery+RT^*^ with complete/partial response	4 (14%) 5 (18%) 10 (36%) 6 (21%) 3 (11%)

* *RT, radiation therapy*.

Of the 61 treatment courses with radiological follow-up, it is noted that 8 (13%) experienced complete response, 10 (16%) experienced partial response, 35 (57%) experienced stable disease, and 8 (13%) experienced local progression.

**Figure 1:**
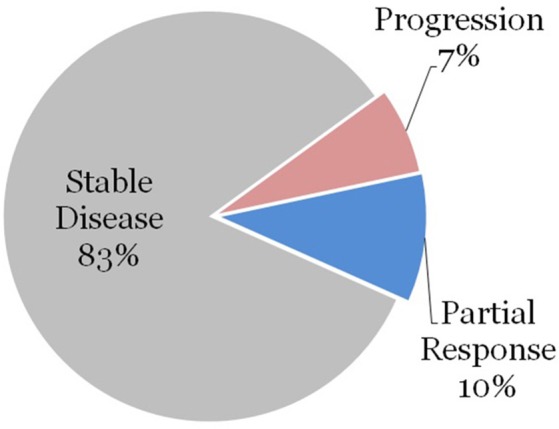
Sites with high-risk features treated with radiation alone.

We further stratified the data by patients who had high-risk features as defined previously. Thirty three (54%) of the treatment courses were for patients with high-risk feature types. Surgery was performed on 3 of these lesions prior to radiation therapy, and these patients achieved a local partial or complete response. The remaining 30 vertebral segments were treated with radiation therapy without surgical excision. None of these patients achieved complete response to radiation therapy alone, 3 lesions (10%) demonstrated partial response, 25 lesions (83%) demonstrated stability, and 2 lesions (7%) demonstrated progression as demonstrated in Figure 1.

**Figure 2:**
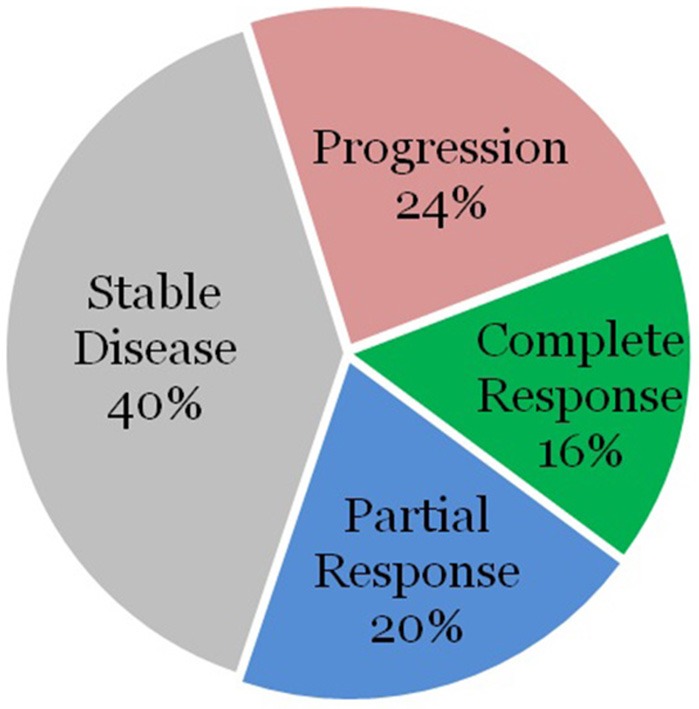
Sites without high-risk features treated with radiation alone.

The remaining 28 patients were without high-risk features. Surgery was performed on 3 of these lesions as well, with all achieving a local partial or complete response. Of the remaining 25 lesions, 4 lesions (16%) demonstrated complete response, 5 lesions (20%) demonstrated partial response, 10 lesions (40%) demonstrated stable disease, and 6 lesions (24%) demonstrated progression as demonstrated in Figure 2.

### Symptomatic Response

Table 3 summarizes symptomatic relief from radiotherapy, including those without radiological follow-up.

**Table 3:** Pain response.

**Response characteristics**	***N* (%)**
Pain relief from radiation therapy (*n* = 98)	
Some degree (pain score decrease ≤ 5)	81 (83%)
Significant (pain score decrease > 5)	33 (34%)

Patients experienced some degree of pain relief after radiotherapy 83% of the time, and had significant relief of pain 34% of the time.

Patients had neurological impairments such as impaired ability to walk, paresthesias, and extremity weakness in 34 of 98 treatments and this was improved with palliative radiotherapy in 28 of the treatments (83%). Specifically, impaired ability to walk was noted prior to 25 of the 98 treatments and this was improved with palliative radiotherapy in 19 of the treatments (76%).

### Additional Analyses of Dose and Relationships

Table 4 summarizes the relationship between radiation dose and radiographic/clinical responses. There was not a statistically significant difference between mean doses administered between the groupings (*p* = 0.74 on a one-way ANOVA).

**Table 4:** Radiological/symptomatic response and dose levels.

**Response characteristics**	**Mean dose [Range]**	***p-*value**
Radiological response (*n* = 61)		*p* = 0.74 (ns)
Complete response Partial response Stable disease Progression	27.2 Gy [20–39.6] 29.6 Gy [20–50] 26.9 Gy [12.5–50] 25.3 Gy [20–30]	
Pain relief from radiation therapy (*n* = 98)		*p* = 0.20 (ns)
Some degree (pain score decrease > 1, ≤ 5) Significant (pain score decrease > 5)	24.0 Gy [16–50] 26.0 Gy [18–50]	

No dose response was noted for the clinical response of all patients (*p* = 0.20 on a two-sample, two-tailed *t*-test).

## Discussion

This is a contemporary cohort study of patients undergoing palliative radiotherapy to the mobile spine using conventional radiation techniques to various doses and using various fractionation schemes in patients undergoing systemic therapy. We were able to identify good clinical symptom relief in the majority of patients but failed to correlate that to radiographic responses, in particular to those patients with high-risk features. The radiographic response was 10% in patients with high-risk features who received radiation alone, despite the wide variety of radiation doses and schemes employed. Most patients experienced some form of pain response, with a subset experiencing a significant pain response. Further, many patients with neurological deficits, including impairment of the ability to walk, were palliated with radiotherapy, including many with high-risk features.

These results question whether a radiographic response is required when treating Multiple myeloma patients with or without high-risk features. In plasmacytoma, it has been demonstrated that doses 45 Gy and higher (10) are needed to achieve a sufficient response and in multiple myeloma for cord compression longer course radiotherapy has been suggested and associated with better functional outcomes than short course RT (11). Nevertheless, in high-risk lesions or in those for whom a significant mass reduction would be considered desirable, we were able to demonstrate symptomatic relief. Therefore, is an increased dose or alternative fractionation schedules such as stereotactic body radiation therapy (SBRT) necessary? As compared to some of these older studies, the majority of our patients also received systemic therapies that may also be contributing to symptomatic relief.

Weakness of this study include the inherent biases associated with retrospective reviews and incomplete long-term follow-up. In addition, there was no consistent radiation dose, fractionation scheme or field size criteria used in this cohort. Lastly, given the discordance between symptom relief and radiographic response, longer and more comprehensive follow-up is needed in this patient population to determine if radiologic response is a good surrogate for measuring outcomes.

Others have explored the use of SBRT for patients with good performance status in a similar patient cohort with promising results (12–14). While fewer fractions may be desirable in a palliative setting, the use of SBRT nonetheless requires further study at least until the best desirable endpoint measure is known.

In summary, this retrospective review of patients who received palliative radiotherapy with a varying degree of doses and fractionation demonstrated good symptomatic response relief but variable radiological response. Patients with high-risk lesions demonstrated less of a radiological response with conventional radiotherapy alone. Further follow up is necessary to identify the best measurable outcome as a means of determining if radiologic response is key to long term symptom relief.

## Data Availability

The datasets generated for this study are available on request to the corresponding author.

## Ethics Statement

This study was carried out in accordance with the recommendations of Northwell Institutional Review Board (IRB) and Cancer Services Scientific Review Committee (CSSRC). The protocol was approved by the Cancer Services Scientific Review Committee (CSSRC) at Northwell. We received a waiver of HIPAA authorization/informed consent from the above board/committee as this was a chart/retrospective review.

## Author Contributions

DM, RM, and MG contributed conception and design of the study. DM, PG, and RM organized the database. DM performed statistical analysis and wrote the first draft of the manuscript. DM, PG, and MG wrote sections of the manuscript. All authors contributed to manuscript revision, read, and approved the submitted version.

### Conflict of Interest Statement

The authors declare that the research was conducted in the absence of any commercial or financial relationships that could be construed as a potential conflict of interest.
